# In vitro characterization of *Haemonchus contortus* trehalose-6-phosphate phosphatase and its immunomodulatory effects on peripheral blood mononuclear cells (PBMCs)

**DOI:** 10.1186/s13071-021-05115-4

**Published:** 2021-12-20

**Authors:** ZhaoHai Wen, XinRan Xie, Muhammad Tahir Aleem, Kalibixiati Aimulajiang, Cheng Chen, Meng Liang, XiaoKai Song, LiXin Xu, XiangRui Li, RuoFeng Yan

**Affiliations:** 1 MOE Joint International Research Laboratory of Animal Health and Food Safety, College of Veterinary Medicine, Nanjing Agricultural University, Nanjing, 210095 Jiangsu People’s Republic of China; 2 State Key Laboratory of Pathogenesis, Prevention and Treatment of High Incidence Diseases in Central Asia, Xinjiang Medical University, Urumqi, 830011 Xinjiang People’s Republic of China

**Keywords:** *Haemonchus contortus*, Trehalose-6-phosphate phosphatase, PBMCs, Immunomodulation

## Abstract

**Background:**

Trehalose-6-phosphate phosphatase (TPP6) is a key enzyme in the trehalose biosynthesis pathway. The accumulation of TPP6 inside the body is harmful to the pathogen, but almost nothing is currently known about the function of TPP6 from *Haemonchus contortus* (CRE-GOB-1).

**Methods:**

The *H. contortus* CRE-GOB-1 (HcGOB) gene was cloned and recombinant protein of GOB (rHcGOB) was expressed; transcription of the HcGOB gene at different developmental stages of *H. contortus* was then studied. The spatial expression pattern of the HcGOB gene in adult female and male worms was determined by both quantitative real-time PCR (qPCR) and immunofluorescence. The binding of the rHcGOB protein to goat PBMCs was assessed by immunofluorescence assay. The immunomodulatory impacts of rHcGOB on cell proliferation, nitric oxide generation and cytokine secretion were assessed by co-culture of rHcGOB protein with goat PBMCs.

**Results:**

The HcGOB protein was transcribed in eggs, infective third-stage larvae (iL3s) and adults of *H. contortus*, with the highest transcript levels found in the egg stage. The transcript levels were significantly elevated in iL3s after manual desheathing. HcGOB was widely distributed in adult worms where it was mainly localized in the gut and gonads. rHcGOB was observed to bind to PBMCs and also to be recognized by sera collected from a goat infected with *H. contortus*. rHcGOB significantly activated the interleukin-10/transforming growth factor β/signal transducer and activator of transcription 3 (IL-10/TGF-β/STAT3) pathway in PBMCs while suppressing the transcription and expression of IL-4 and IL-17.

**Conclusions:**

These results suggest that the HcGOB gene plays an important role in the development, parasitism and reproduction of *H. contortus*. The rHcGOB protein affected the immunomodulatory function of PBMCs in the in vitro study, suggesting that this protein would be a promising vaccine target.

**Graphical Abstract:**

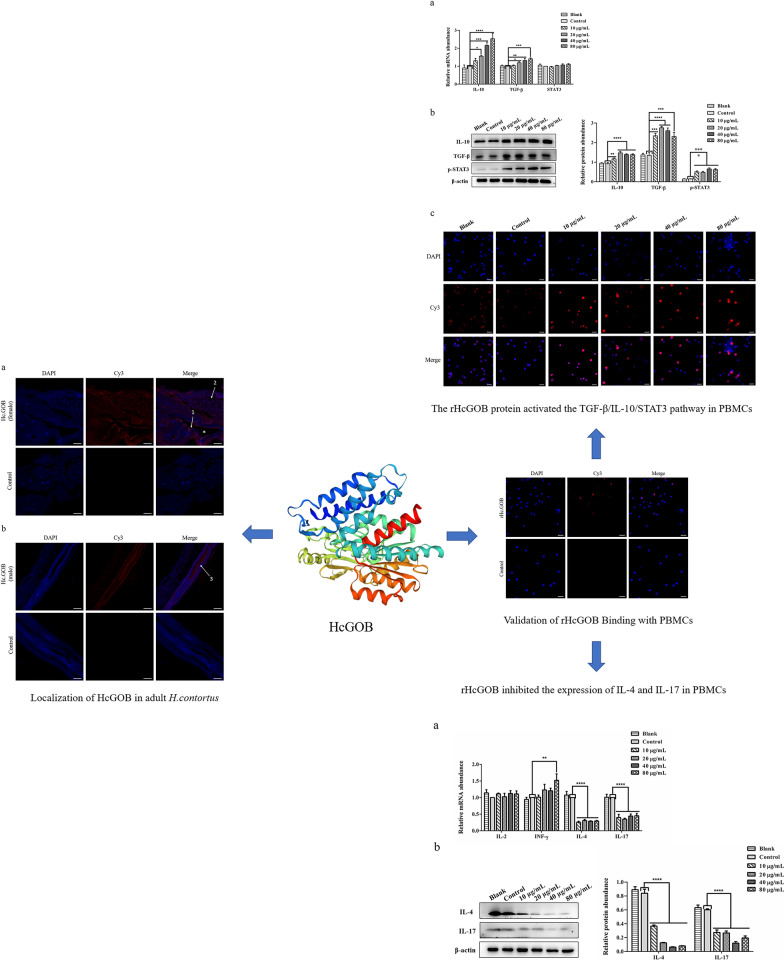

**Supplementary Information:**

The online version contains supplementary material available at 10.1186/s13071-021-05115-4.

## Background

*Haemonchus contortus* is an important gastrointestinal nematode of ruminants in tropical and subtropical regions of the world. It is a major health burden in sheep and causes significant economic losses to farmers [1, 2]. The control of animal parasitic nematodes is currently almost entirely dependent on anthelmintics [2], but heavy use [3] has resulted in the development of parasite resistance against these anthelmintic drugs, threatening the capability to control parasitic infections. Vaccines offer an attractive alternative strategy for the control of haemonchosis [1], but both the extensive genetic diversity and the complicated immunomodulatory properties of *H. contortus* hinder the development of a suitable vaccine [1, 4]. With the exception of the first commercial vaccine in Australia, Barbervax® (Wormvax Australia Pty Ltd., Albany, WA, Australia), which achieved successful immunization by using native *H. contortus* antigens [5–8], only a few studies have been conducted that focus on the protective efficacy of *H. contortus* subunit vaccines [9].

The coexistence of nematodes with their hosts relies on a complex regulatory relationship referred to as the nematode–host immune system [10, 11]. Previous studies have shown that the excreted secretory proteins (ESPs) of *H. contortus* can inhibit the functions of peripheral blood mononuclear cells (PBMCs) in vitro, thereby inhibiting cell proliferation and nitric oxide (NO) secretion [12]. The activation of *H. contortus* excretory/secretory products (HcESPs) exercises a critical control on the production of T-cell cytokines, primarily by promoting the secretion of interleukin (IL)-10, IL-17A and transforming growth factor beta 1 (TGF-β1) and by inhibiting the production of IL-2, IL-4 and interferon gamma (IFN-γ) [13]. Thus, understanding how the mechanisms of *H. contortus* regulate the host immune response by promoting coexistence with the host may help towards the development of novel preventive strategies against haemonchosis.

Trehalose is a natural disaccharide synthesized by a variety of different organisms, such as bacteria, yeast, nematodes, insects and plants, but it is not present in vertebrates [14, 15]. There have been some hints that trehalose also has a specific bioprotective property [16–21] and is capable of providing protection to organisms in harsh environments, especially those found in a high temperature zone, freezing zone, high osmotic pressure zone, highly acidic environment and areas with toxic substances as well as in drought condition. Trehalose is the main blood sugar in insects and functions as a source of energy to maintain insect flight [22]. It also has been identified in the eggs of the nematode *Ascaris lumbricoides*, accounting for up to 8% of the dry weight of the eggs [23], and in the larvae of the parasitic ascarid *Porrocaecum decipiens* (Nematoda), accounting for up to 6% of dry weight [24]. Trehalose-6-phosphate phosphatase (TPP6/GOB) is a key enzyme in the synthesis of trehalose [25, 26], catalyzing the hydrolysis of trehalose-6-phosphate to trehalose and phosphate [27]. It has been shown that the accumulation of trehalose-6-phosphate induces toxicity in pathogens [25, 28, 29]. The conserved nature of the binding residues of TPP6 in pathogenic nematodes and bacteria favors TPP6 as a suitable target for a wide range of novel anthelmintic and antibacterial drugs [30]. These data suggest that trehalose plays an important role in the development of nematodes, but reports on the role of trehalose in the development, parasitism and other life activities of *H. contortus* are lacking. Here we report our study of the trehalose synthesis pathway and its key enzymes, with the aim to provide a basis for finding new drug targets against haemonchosis. The findings may also identify a potential target for the development of a vaccine against *H. contortus*. The overal aim of this study was to investigate the expression and localization of the GOB protein in *H. contortus* and to elucidate the interaction of the recombinant protein of GOB (rHcGOB) with host PBMCs.

## Methods

### Animals and parasites

Locally crossbred goats aged 6–10 months were housed indoors with access to biofeed and free access to an area of drinking water. Infective third-stage larvae (iL3s) of *H. contortus* were isolated from feces of single-species infected goats, as previously described [12, 31]. Each goat was orally infected with 8000 iL3s. To confirm infection, fecal samples were collected and checked weekly for the presence of *H. contortus* eggs. Male and female adult parasites and eggs were collected as previously described [31, 32]. Healthy goats were kept separately in the ventilated cages to prevent accidental infection from nematodes and fed with hay and whole-shell corn; all goats had free access to fresh water in their enclosures.

Wistar rats (body weight 150–200 g) were procured from the Experimental Animal Center of Jiangsu, China (quality certificate: SCXK 2008-0004) and raised in the Laboratory of Animal Centre, Nanjing Agricultural University China. They were provided with sterilized water and food.

### Molecular cloning of HcGOB

Adult *H. contortus* worms were obtained as previously described [33]. Total RNA was extracted from adult worms by the TRIzol reagent (Invitrogen, Carlsbad, CA, USA) method [34]. The complementary DNA (cDNA) was synthesized by HiScript III Kit (Vazyme, Nanjing, China) according to the manufacturer’s instructions. Based on the sequences of *H. contortus* (GenBank: HF967182.1), the HcGOB gene was amplified by specific primers (GOB-F and GOB-R, Additional file 1: Table S1). PCR assays were performed in a total reaction volume of 50 μl [25 μl 2× primer master mix (Takara, Dalian, China), 19 μl ddH2O, 2 μl cDNA, 2 μl of each primer) and amplification was conducted according to the kit instructions, as described by Aleem et al. [35]. The gene product was purified by using the Gel Extraction Kit (Vazyme, Nanjing, China) and ligated into the expression plasmid pET28a(+) vector (Novagen, Madison, WI, USA). The recombinant plasmid, pET28a (+)/HcGOB, was identified through digestion by restriction enzymes (*Bam*HI and* Hin*dIII), and the sequence was analyzed with online BLAST analysis.

### Bioinformatic, phylogenetic analyses and molecular modeling

Nucleotide and amino acid sequences were aligned using the BLAST program (https://blast.ncbi.nlm.nih.gov/Blast.cgi). The inferred HcGOB amino acid sequences were aligned with homologous sequences belonging to nine other species, namely *Ancylostoma caninum*, *Ancylostoma ceylanicum*, *Caenorhabditis elegans*, *Dictyocaulus viviparus*, *Haemonchus placei*, *Oesophagostomum dentatum*, *Necator americanus*, *Teladorsagia circumcincta* and *Toxocara canis*. The phylogenetic tree of HcGOB and TPP6 homologs were constructed using the neighbor-Joining (NJ) method in MEGAx software. Moreover, we forecast the three-dimensional pattern of homo-dimer by using homology modeling (https://swissmodel.expasy.org/interactive/HnydV2/templates/). The signal peptide was predicted by the SignalP 4.1 Server (http://www.cbs.dtu.dk/services/SignalP/), the transmembrane zone was predicted by the TMHMM Server v. 2.0 (http://www.cbs.dtu.dk/services/TMHMM/) and probable N-glycosylation sites were predicted with the NetNGlyc 1.0 Server (http://www.cbs.dtu.dk/services/NetNGlyc/).

### Expression and purification of the rHcGOB protein

The rHcGOB protein was purified and expressed as previously described [36, 37]. The recombinant plasmid [pET28a(+) ligated with GOB] was transformed into *Escherichia coli* BL21(DE3) and cultured into Luria–Bertani medium containing kanamycin at 37 °C. When the cultured cells reached the logarithmic phase (OD600; approx. 0.6), isopropyl-β-d-thiogalactopyranoside (IPTG; working concentration: 1 mM) was added and the medium cultured for a further 5 h at 37 °C with stirring (180 rpm). After induction of the expression, the culture was centrifuged at 7104 *g* for 15 min and the supernatant discarded; the bacteria pellet was then washed twice with phosphate-buffered saline (PBS) and resuspended in supernatant binding buffer. The process of freezing and thawing was repeated 2–3 times to facilitate breaking down of the bacterial cells, followed by ultrasound-assisted centrifugation of the lysate (7104 g at 4 ℃ for 15 min) and collection of the supernatant. The precipitate was dissolved overnight with 25 ml binding buffer at 4 ℃. Expression of the rHcGOB protein in the supernatant and precipitate was analyzed by electrophoresis. The recombinant HcGOB protein was purified by passage through a Ni2^+^-nitrilotriacetic acid (Ni-NTA) column. The lipopolysaccharides from the recombinant proteins were detoxified using the ToxinEraserTM Endotoxin Removal kit (GenScript, Nanjing, China).

### Preparation of polyclonal antibodies

The purified rHcGOB protein (250 µg) was mixed with Freund’s complete adjuvant (1:1; Sigma-Aldrich, St. Louis, MO, USA) and injected subcutaneously into Wistar rats. Two weeks following injection of the initial dose, the rats were injected with the same dose of the rHcGOB protein mixed with incomplete Freund’s adjuvant (1:1; Sigma-Aldrich, Shanghai), followed by a second dose 7 days later. On day 7 following the last immunization, sera were obtained and stored at −80 °C until further use.

### Detection of rHcGOB and native HcGOB protein by western blot assay

The rHcGOB protein and soluble proteins of adult *H. contortus* were transferred to polyvinylidene difluoride (PVDF) membranes (MilliporeSigma, Burlington, MA, USA) and then subjected to 12% sodium dodecyl sulfate-polyacyrlamide gel electrophoresis (SDS-PAGE) for western blotting analysis, as described previously [38]. Nonspecific binding was blocked with 5% skim milk in Tris-buffered saline containing 0.1% Tween-20 (TBST). The membranes were washed three times with TBST and incubated with primary antibody (serum from goat infected with *H. contortus* and rat-anti-rHcGOB antiserum,1:100 dilution in TBST) at 4 °C overnight, following which the membranes were washed again three times and incubated with horseradish peroxidase (HRP)-conjugated rabbit anti-goat IgG/rabbit anti-rat IgG (diluted 1:5000 in TBST) for 1 h at 37 °C. Finally, the Tanon™ High-sig ECL Western Blotting Substrate Kit (Tanon, Shanghai, China) was used to detect bound antibodies according to the manufacturer's instructions.

### Assessing HcGOB transcription by quantitative real-time PCR

The L3s were treated with 0.1% sodium hypochlorite at 38 °C for approximately 30 min to remove the sheaths; most of the detached sheaths were visible under the microscope. The L3s were then washed with PBS to obtain exsheathed L3s (xL3s) [32]. Transcription of the HcGOB gene was examined in each of the developmental stages (eggs, L3s, xL3s and adults) and both sexes (males and females) of *H. contortus* by quantitative real-time PCR (qPCR) assay using the primers HcGOB-F and HcGOB-R (Additional file 1: Table S2). Total RNA was isolated separately from eggs (about 1 × 10^7^), L3s (about 1 × 10^5^), xL3s (about 1 × 10^5^), adult females (100 mg) and adult males (100 mg) using the TRIzol technique (Vazyme Biotech, Nanjing, Jiangsu, China) according to the manufacturer’s protocol. Then 1 μg RNA of each stage was used to make the first-strand cDNA, synthesized by the HiScript III cDNA Synthesis Kit (Vazyme, Nanjing, China) according to the manufacturer’s instructions. The β-tubulin gene was used as the reference gene [39]. The qPCR conditions were: 1 cycle at 95 °C/30 s; 40 cycles at 95 °C/10 s, 60 °C/30 s. The dissociation curve was generated under the following conditions: 95 °C/15 s, 60 °C/1 min and 95 °C/15 s. The data were analyzed according to raw cycle thresholds (Ct) which were obtained using ABI Prism 7500 software (Applied Biosystems, Foster City, CA, USA) using the comparative delta–delta Ct (2^−ΔΔ Ct^) method.

### Localization assay

The native HcGOB protein in adult *H. contortus* worms (both female and male) was examined by immunofluorescence assay (IFA).

*Haemonchus contortus* adult worms were removed from the abomasum of the goat, fixed with 4% paraformaldehyde and cut into 4-µm-thick sections on a rotary microtome (Leica Microsystems GmbH, Wetzlar, Germany). The sections were placed on microscope slides and treated with 0.01 mol/l citrate buffer to repair the antigen and non-specific binding was blocked with 5% skim milk, for 30 min at 37 °C, following which the slides were incubated with the primary antibody (rat anti-rHcGOB antiserum and normal rat serum) at 4 °C overnight. The slides were then washed 5 times (10 min each) with PBS containing 0.1% Tween 20 (PBST) buffer and incubated with the secondary antibody (goat anti-rat IgG antibody) conjugated to sulfo-cyanine 3 (Cy3; 1:500 dilution; Beyotime, Shanghai, China) for 1 h at 37 °C, following which the slides were washed 5 times (10 min each) with PBST and the nuclei stained with 4′,6-diamidino-2-phenylindole (DAPI; Beyotime). As a last step, the slides were washed 5 times and the sections observed by laser confocal scanning microscopy (model LSM 710; Carl Zeiss Microscopy, Jena, Germany).

### Separation of PBMCs

The PBMCs were separated from goat blood samples by ordinary Ficoll-Paque density gradient centrifugation (GE Healthcare, Chicago, IL, USA), as previously described [40]. The PBMCs were washed twice with PBS (pH 7.4) and adjusted to the desired density (1 × 10^6^ cell/ml) in the RPM 1640 medium containing 10% fetal bovine serum, 100 U/ml streptomycin, and 100 mg/ml penicillin (UK Gibco, Paisley, UK). The trypan blue ejection test was used to evaluate cell viability [41].

### PBMC binding assay

The binding ability of rHcGOB protein to goat peripheral blood mononuclear cells was detected by IFA as previously described [33, 42, 43], . The fresh goat PBMCs were inoculated with 10 μg/ml rHcGOB or PBS in a 24-well plate (1 ml/well) and the plate incubated at 37 °C with 5% CO_2_ for 2 h. The cells were then washed with PBS, placed on poly l-lysine-coated slides for 20 min and fixed with 4% phosphate-buffered paraformaldehyde at room temperature. The slides were then treated with 5% bovine serum albumin (BSA) at 37 °C for 1 h, then incubated with primary antibody (rat anti-rHcGOB serum, 1:100 diluted) at 4 °C overnight, subsequently washed 3 times with PBST and incubated in the dark for 1 h with goat anti-rat IgG antibody conjugated with Cy3 (Beyotime, 1: 500 dilution) at 37 °C. DAPI was then added and the slides were again incubated in the dark at room temperature for 10 min. The binding of PBMCs to rHcGOB protein was observed by laser confocal scanning microscopy (model LSM 710; Carl Zeiss Microscopy).

### NO production assay

Secretion of NO by cells was detected as described previously [36]. In short, the separated goat PBMCs (1 × 10^6^ cells/ml) were first washed with PBS and then 100 μl of the cells in DMEM culture medium was poured into a 96-well plate, with wells containing different concentrations of rHcGOB (10, 20, 40, and 80 μg/ml) and PBS (negative control), and incubated at 37 °C in a humidified atmosphere (5% CO_2_ for 24 h). In accordance with the instructions of the Total Nitric Oxide Analysis Kit (Beyotime), NO production was analyzed in triplicate by the Griess method. The absorbance of colored solution was measured on a microplate reader at OD_540_.

### Cell proliferation assay

The cell proliferation test was carried out in triplicate using Cell Counting Kit-8 (CCK-8) analysis reagent (Beyotime), as described previously [44, 45]. Briefly, fresh goat PBMCs (1 × 10^6^ cells/ml) were seeded into a 96-well plate and incubated with rHcGOB protein (10, 20, 40, and 80 μg/ml) in a humidified atmosphere (at 37 °C with 5% CO2 for 72 h). Before measuring absorbance (OD_450_) with a miniature flat panel reader, 10 μl of CCK-8 reagent was added to each well and incubated at 37 °C for 4 h.

### Transcriptional abundance of cytokines and related pathway molecules were detected by qPCR

The group settings were the same as those for the NO secretion assays. IL-2, IFN-γ, IL-4, IL-10, IL-17, TGF-β1 and signal transducer and activator of transcription 3 (STAT3) transcription levels were detected by qPCR. Total RNA was extracted by the TRIzol method according to the manufacturer’s instructions (Invitrogen) and cDNA was synthesized using the HiScript III Kit (Vazyme, Jiangsu Nanjing, China). The transcriptional levels of mRNA were detected by qPCR, and the β-actin gene was used as the reference gene. In accordance with a previous study [46], the primer sequences shown in Additional file 1: Table S2 were used. The calculation was carried out according to the comparative Ct (2^−ΔΔCt^) method.

### Detection of cytokine and related pathway molecule expression by western blot assay

The group settings were the same as those used for the NO secretion assays. Protein concentration was measured using a BCA Protein Determination Kit (Beyotime). The protein (30 μg) was separated by SDS-PAGE and transferred onto the PVDF membrane. After blockage of non-specific binding with 5% skim milk or BSA for 2 h at room temperature, the membranes were incubated with primary antibodies (TGF-β1 monoclonal antibody 1:500; Santa Cruz Biotechnology, Inc., Dallas, TX, USA), IL-4, IL-10, IL-17 and P705-STAT3 polyclonal antibody (1:1000; Affinity Biosciences, Jiangsu province, China), at 4 °C overnight, then washed with TBST. The washed membranes were incubated with secondary antibodies (anti-goat IgG, anti-rat IgG, anti-mouse IgG, anti-rabbit IgG;1:8000; Beyotime) conjugated with HRP. The expression of β-actin was evaluated using an anti-β-actin antibody (1:10000) as an internal quantitative control. The membranes were washed in TBST and stained with the Tanon™ High-sig ECL Western Blotting Substrate Kit (Tanon, Shanghai, China). The relative protein expression levels were analyzed using Image J software.

### Detection of nuclear translocation of phosphorylated STAT3 by IFA

The group settings were the same as those used for the NO secretion assays. IFA was performed using STAT3 polyclonal antibody (1: 50; Affinity Biosciences) as primary antibody and goat anti-rabbit IgG conjugated with Cy3 (Beyotime; 1:500 dilution) as a secondary antibody. The procedure was the same as that for the PBMC binding assay.

### Data analysis

Statistical analysis was performed using the GraphPad Premier 6.0 software package (GraphPad Prism; GraphPad Software Inc., San Diego, CA, USA ). The results were presented as the mean ± standard error of the mean (SEM). Student’s t-test was performed to determine differences between the two groups. The differences between groups determined one‐way analysis of variance (ANOVA), with statistical significance set at *P < 0.05, **P < 0.01, ***P < 0.001 and ****P < 0.0001, respectively. All experiments were repeated a minimum of three times.

## Results

### Cloning, expression and purification of HcGOB

**Fig. 1:**
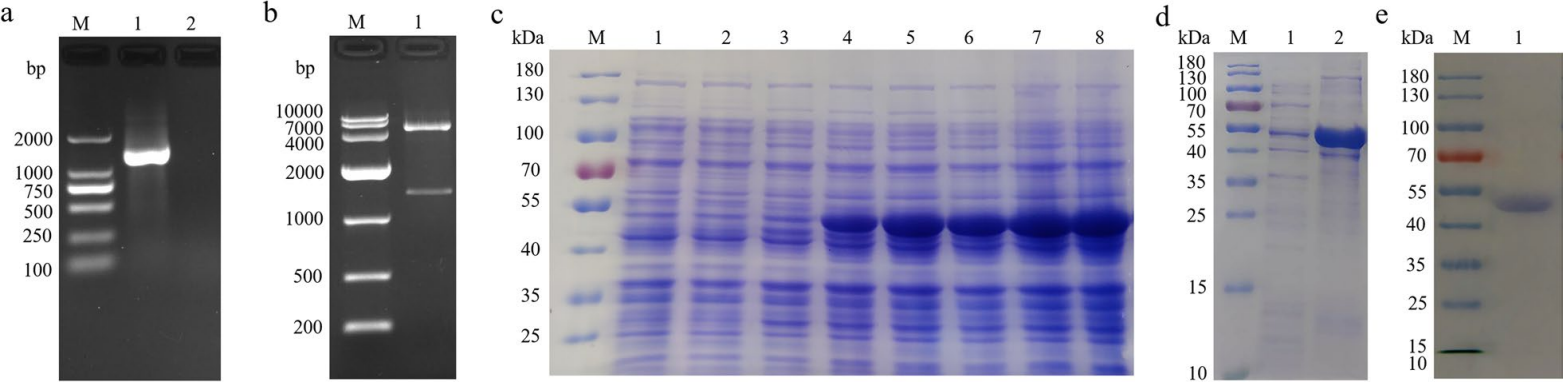
Cloning, expression and purification of HcGOB**. a** Amplification of HcGOB. Lanes: M, DNA marker DL 2000; 1, amplification products of the GOB gene; 2, negative control. **b** Lanes: M, DNA marker DL 10000; 1, digestion of pET-28a/HcGOB by enzymes. **c**–**e** Expression and purification of HcGOB. Lane M, standard protein molecular weight marker. **c** Lanes: 1, 2, pET-28a induced by IPTG for 0 and 5 h, respectively; 3–8, pET-28a/HcGOB induced by IPTG for 0, 1, 2, 3, 4 and 5 h, respectively. **d** Lanes: 1, supernatant of expression products; 2, inclusion body of expression products. **e** Lane 1: purification of recombinant (r) HcGOB

Using the cDNA of adult *H. contortus* worms as a template, the HcGOB gene was amplified using primers GOB-F and GOB-R, and the size of the amplified band was about 1299 bp (Fig. 1a). The HcGOB gene was successfully cloned into the pET28a expression vector, as verified by enzymatic digestion of the 1299-bp amplified band (Fig. 1b). The recombinant plasmid (pET28a/HcGOB) was induced by IPTG and expressed in *E. coli* BL21 (DE3). Following separation by SDS-PAGE, staining with Coomassie Brilliant Blue revealed fusion protein rHcGOB (Fig. 1c). The rHcGOB protein was expressed in the form of inclusion bodies (Fig. 1d) and purified by passage through the Ni-NTA super chromatography column. SDS-PAGE showed that the molecular weight of the purified recombinant protein was approximately 52 kDa (Fig. 1e), as expected.

### Sequence and phylogenetic analysis of HcGOB

**Fig. 2:**
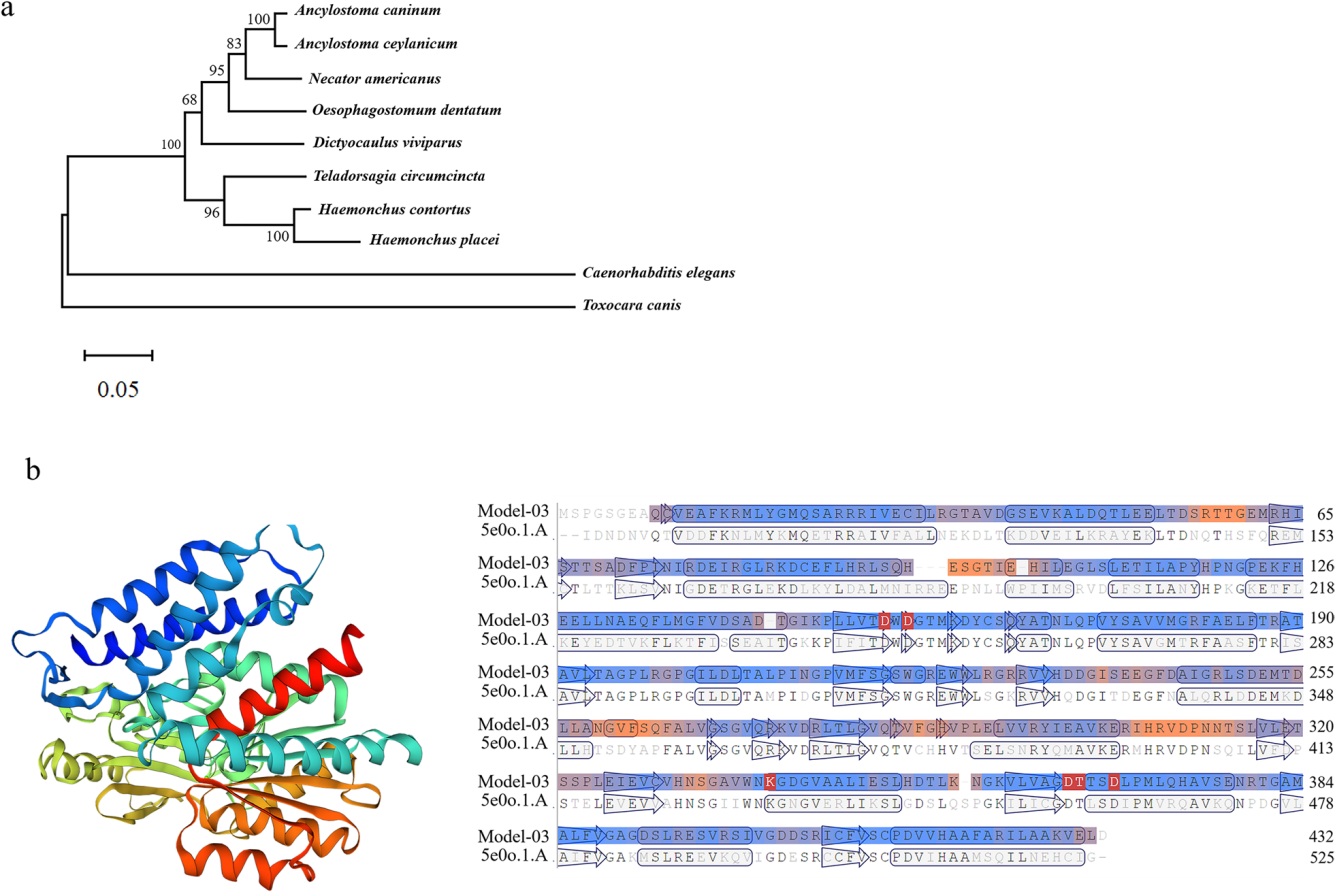
Bioinformatic and phylogenetic analyses.** a** Phylogenetic relationship of *Haemonchus contortus* HcGOB (CRE-GOB-1) with other TPP6 from selected species. The phylogenetic tree was constructed using amino acid sequences of HcGOB and the TPP6 homologs from other nematode species, including *Ancylostoma caninum*, *Ancylostoma ceylanicum*, *Caenorhabditis elegans*, *Dictyocaulus viviparus*, *Haemonchus placei*,* Oesophagostomum dentatum*, *Necator americanus*,* Teladorsagia circumcincta* and *Toxocara canis*. The tree was constructed using the neighbour-joining method in MEGA software. Bootstrap values above or below the branches are shown for robust clades. **b** Trehalose-phosphatase. Structure of unliganded trehalose-6-phosphate phosphates from *Brugia malayi*. The predicted *H. contortus* HcGOB three-dimensional model shows 56.6% identity with the TPP6 from *Brugia malayi*

The isolated sequences of the HcGOB gene were confirmed by BLAST and the protein sequence was translated by DNASTAR software (DNASTAR, Madison, WI, USA) as 432 amino acids residues. Multiple sequence alignment of HcGOB with available homologous sequences on the NCBI database and UniProt database revealed that HcGOB was closely related to the GOB of *Ancylostoma caninum* (84.4%), *Ancylostoma ceylanicum* (79.0%), *Caenorhabditis elegans* (59.7%), *Dictyocaulus viviparus* (78.4%), *Haemonchus placei* (85.1%), *Oesophagostomum dentatum* (75.7%), *Necator americanus* (78.1%), *Teladorsagia circumcincta* (90.0%) and *Toxocara canis* (60.9%). The trees constructed using the NJ (neighbor-joining), ML (maximum likelihood) and MP (maximum parsimony) methods showed a similar topography. HcGOB had the closest relationship with the TPP6 of *H. placei* (100% nodal support) and *T. circumcincta* (96% nodal support) (Fig. 2a). The SWISSMODEL server provided the best template to predict the HcGOB three-dimensional model and TPP6 from *Brugia malayi* (PDB: 5e0o.1.A) with 56.6% identity (Fig. 2b). No signal peptide and transmembrane structure were found in the inferred protein sequence (Additional file 2: Figures S1, S2), but an N-glycosylation modification site was present (Additional file 2: Figure S3).

### Western blot analysis of native HcGOB and rHcGOB

**Fig. 3:**
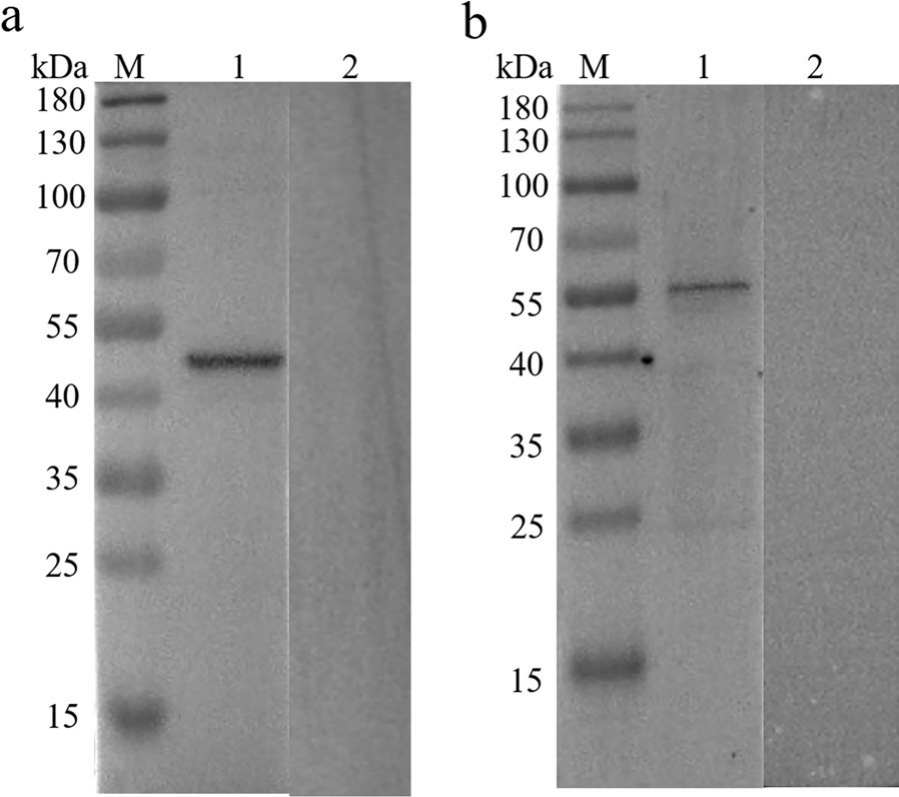
Western blot identification of recombinant (r) HcGOB protein (**a**) and adult *H. contortus* native HcGOB protein (**b**). **a** Lanes: M, Standard protein molecular weight marker; 1, rHcGOB detected when incubated with serum from *H. contortus*-positive goat; 2, no protein was detected with normal goat sera. **b** Lanes: 1, Native HcGOB protein detected by incubation with anti-rHcGOB rat sera; 2, no protein was detected with normal rat sera

Western blot analysis showed that the rHcGOB protein was recognized by goat serum infected with *H. contortus* (Fig. 3a) and that the native HcGOB protein recognized by anti-rHcGOB polyclonal antibodies (Fig. 3b). However, the molecular weight of adult native GOB protein was slightly higher than that of the prokaryotic rHcGOB, possibly due to post-translational modification of *H. contortus* GOB protein.

### Relative transcript levels of the HcGOB gene at different developmental stages of *H. contortus*

**Fig. 4:**
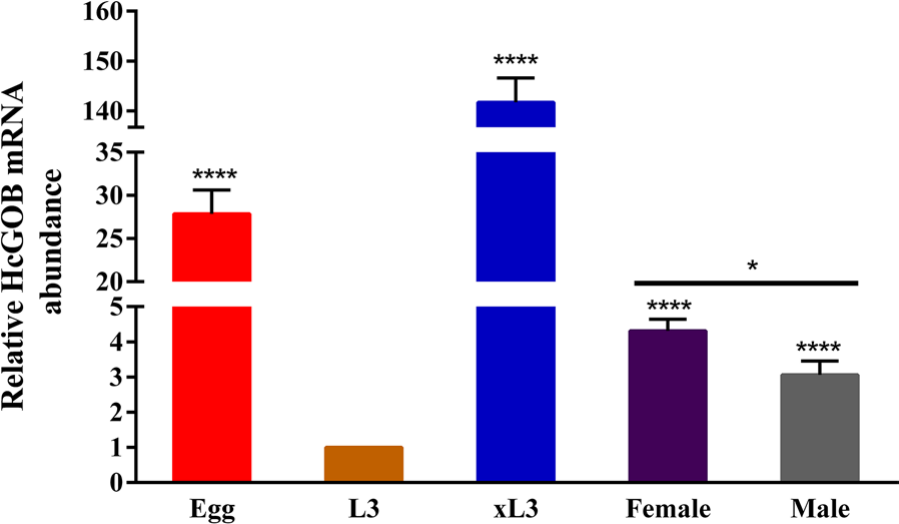
Transcriptional analysis of HcGOB during different developmental stages of *H. contortus*. HcGOB abundance is shown as mean values ± standard error of the mean (SEM) relative to abundance at the L3 stage (L3 = 1). The results are representative of three independent experiments. Asterisks indicate significant differences at ***P < 0.001 and ****P < 0.0001 versus L3 and at *P < 0.05 female versus male adult worms

The transcription of the HcGOB gene at various development stages of *H. contortus* was examined by qPCR using the β-tubulin gene as an internal control. As shown in Fig. 4, the HcGOB gene transcript levels were lowest in the L3 stage and significantly higher in the adult (female: t-test, *t*_(6)_ = 10.52, *P* < 0.0001; male: t-test, *t*_(6)_ = 10.03, *P* < 0.0001), xL3 (t-test, *t*_(6)_ = 28.79, *P* < 0.0001) and egg stages (t-test, *t*_(6)_ = 9.775, *P* < 0.0001). In the adult stage, the incease in HcGOB transcription was significantly higher in female than in male worms (t-test, *t*_(6)_ = 9.775, *P* < 0.0001). This result suggests that the HcGOB protein may play an important biological function in nematode desheathing and embryonic development.

### Localization of HcGOB in adult *H. contortus*

**Fig. 5:**
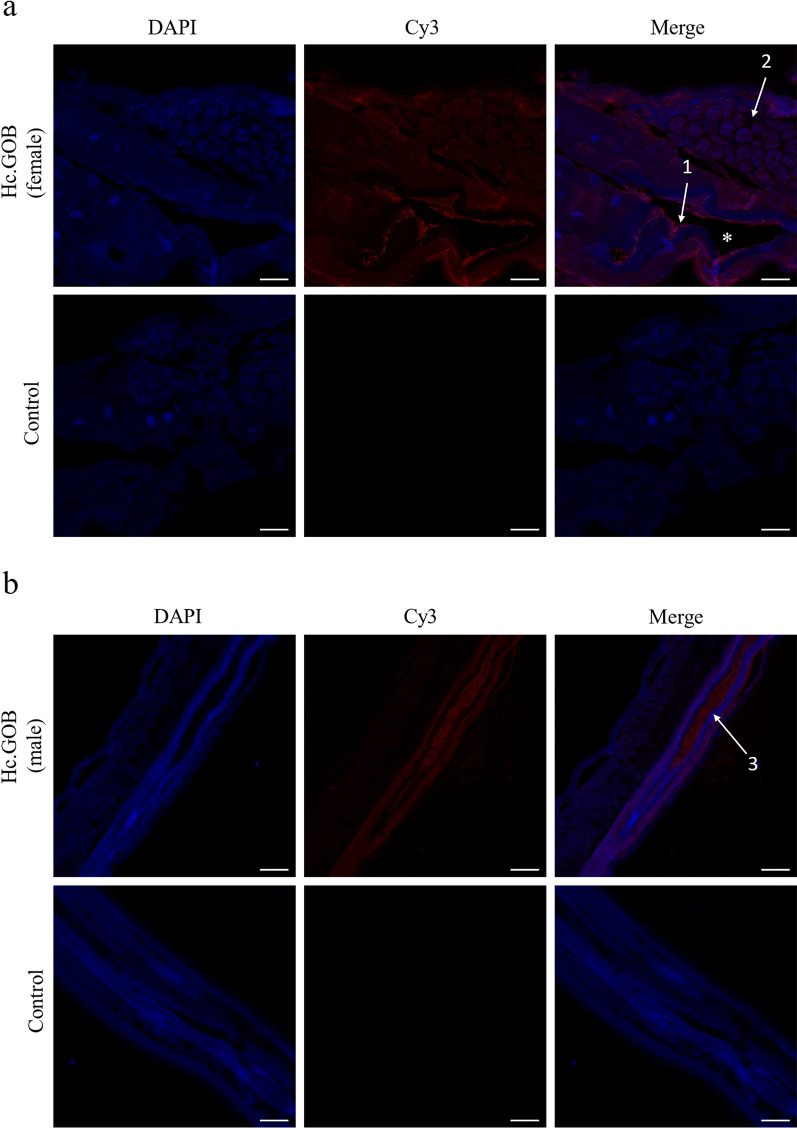
Localization of HcGOB in adult male and female *H. contortus* worms. The red color indicates localization of the target protein stained with Cy3, and the blue color indicates the localization of nuclei stained with DAPI. Merge indicates the merged image of DAPI and Cy3. **a** Targeted HcGOB protein localized in the tissues of female worms, **b** targeted HcGOB protein localized in the tissues of male worms. No red fluorescence was observed in the control. Organs annotated in the Merge image are intestinal microvilli (1), eggs in the uterus (2), cavity of the intestine (*) and intestine (3). Scale bars: 50 μm

The IFA results showed that HcGOB protein was widely expressed in the adult worms of *H. contortus*. In adult females, HcGOB expression was localized mainly on the surface of the intestine, in gonads and in eggs (Fig. 5a). Strong expression was especially shown on the surface of eggs and the intestinal microvilli (Fig. 5a). In adult males, HcGOB was expressed mainly inside the gut and in gonads (Fig. 5b).

### Validation of rHcGOB binding with PBMCs

**Fig. 6:**
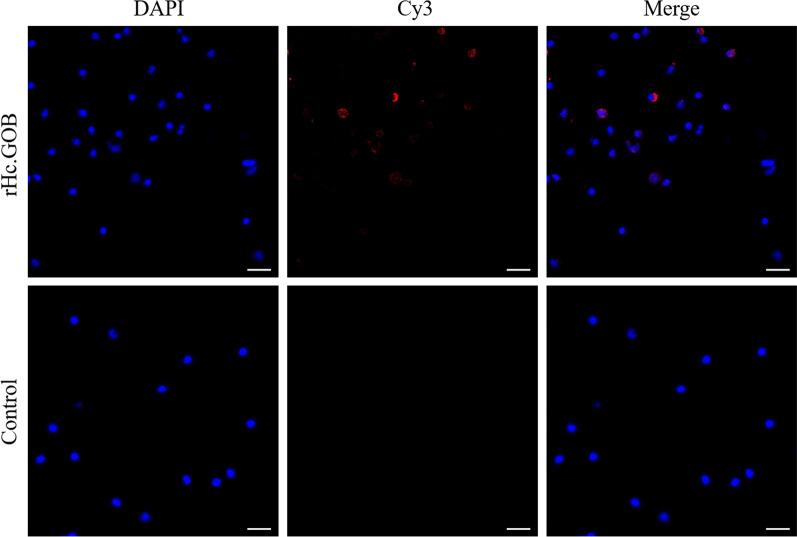
Binding of rHcGOB protein to PBMCs. The red color indicates the localization of target protein stained with Cy3 on PBMCs, and the blue color indicates the localization of nuclei stained with DAPI. Merge indicates the merged image of DAPI and Cy3. No red fluorescence was observed in the control. Scale bars: 20 μm

The IFA confirmed that rHcGOB could bind to goat PBMCs, and the confocal microscopic images showed that rHcGOB protein could bind to the surface of goat PBMCs. As shown in Fig. 6, the nuclei of the cells were stained with blue fluorescence, the target proteins were stained red and the controls showed no fluorescence.

### Effects of rHcGOB protein on proliferation of goat PBMCs

**Fig. 7:**
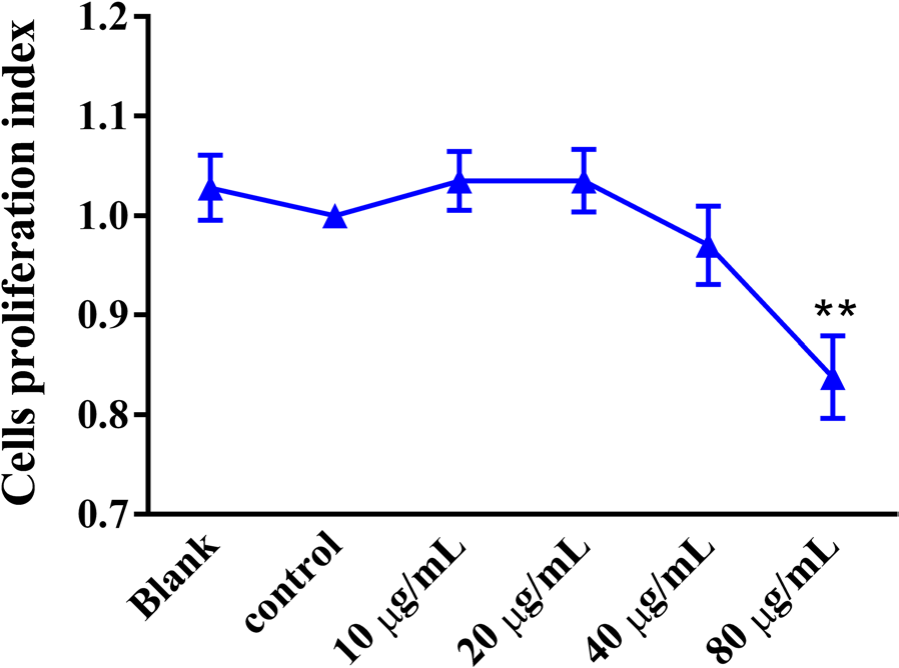
Influence of rHcGOB on PBMC proliferation. Cells were incubated with different concentrations of rHcGOB or with PBS (control) at 37 °C, and 5% CO_2_ for 72 h. The cell proliferation index was determined by setting the OD_450_ values of the control group as 100%. Data are presented as the mean ± SEM from three independent experiments, with three technical replicates per group. Asterisks indicate a significant difference at **P < 0.01 versus the control group

Compared with the control PBMCs (incubated with PBS), incubation with 10, 20 and 40 μg/ml rHcGOB had no significant effect on the proliferation of PBMCs (10 μg/ml: ANOVA, *F*_(5, 12)_ = 6.745, *P* = 0.4163; 20 μg/ml: ANOVA, *F*_(5, 12)_ = 6.745, *P* = 0.4156; 40 μg/ml: ANOVA: *F*_(5, 12)_ = 6.745, *P* = 0.4834). However, treatment with a high concentration (80 μg/ml) of the rHcGOB protein did significantly inhibit the proliferation of PBMCs (16.23% reduction) (ANOVA, *F*_(5, 12)_ = 6.745, *P* = 0.0020), as shown in Fig. 7.

### Effects of rHcGOB protein on NO secretion of goat PBMCs

**Fig. 8:**
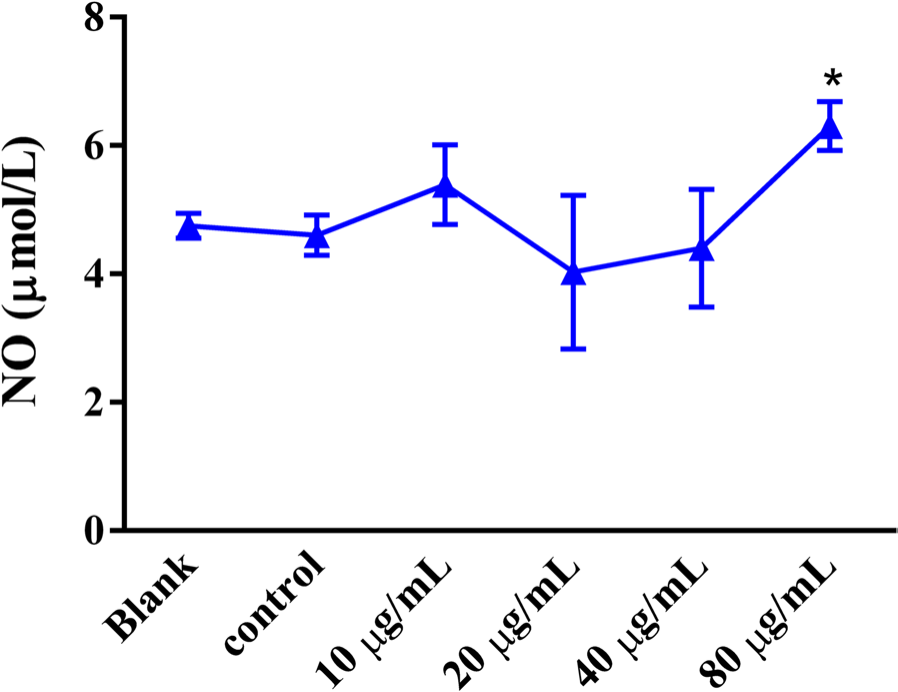
Influence of rHcGOB on NO production by PBMCs in vitro. Cells were incubated with different concentrations of rHcGOB protein or with PBS (control) for 24 h at 37 °C and 5% CO_2_. Data are presented as the mean ± SEM from three independent experiments, with three technical replicates per group. Asterisk indicates a significant difference at *P < 0.05 versus the control group

Compared with the control PBMCs (incubated with PBS), incubation with 10, 20 and 40 μg/ml rHcGOB had no obvious effect on NO secretion in PBMCs (10 μg/ml: ANOVA, *F*_(5, 12)_ = 3.723, *P* = 0.2125; 20 μg/ml: ANOVA, *F*_(5, 12)_ = 3.723, *P* = 0.3536; 40 μg/ml: ANOVA: *F*_(5, 12)_ = 3.723, *P* = 0.7401). However, treatment with a high concentration (80 μg/ml) of rHcGOB protein significantly increased NO secretion (ANOVA: *F*_(5, 12)_ = 3.723, *P* = 0.0149), as shown in Fig. 8.

### The rHcGOB protein activated the IL-10/TGF-β/STAT3 pathway in PBMCs

**Fig. 9:**
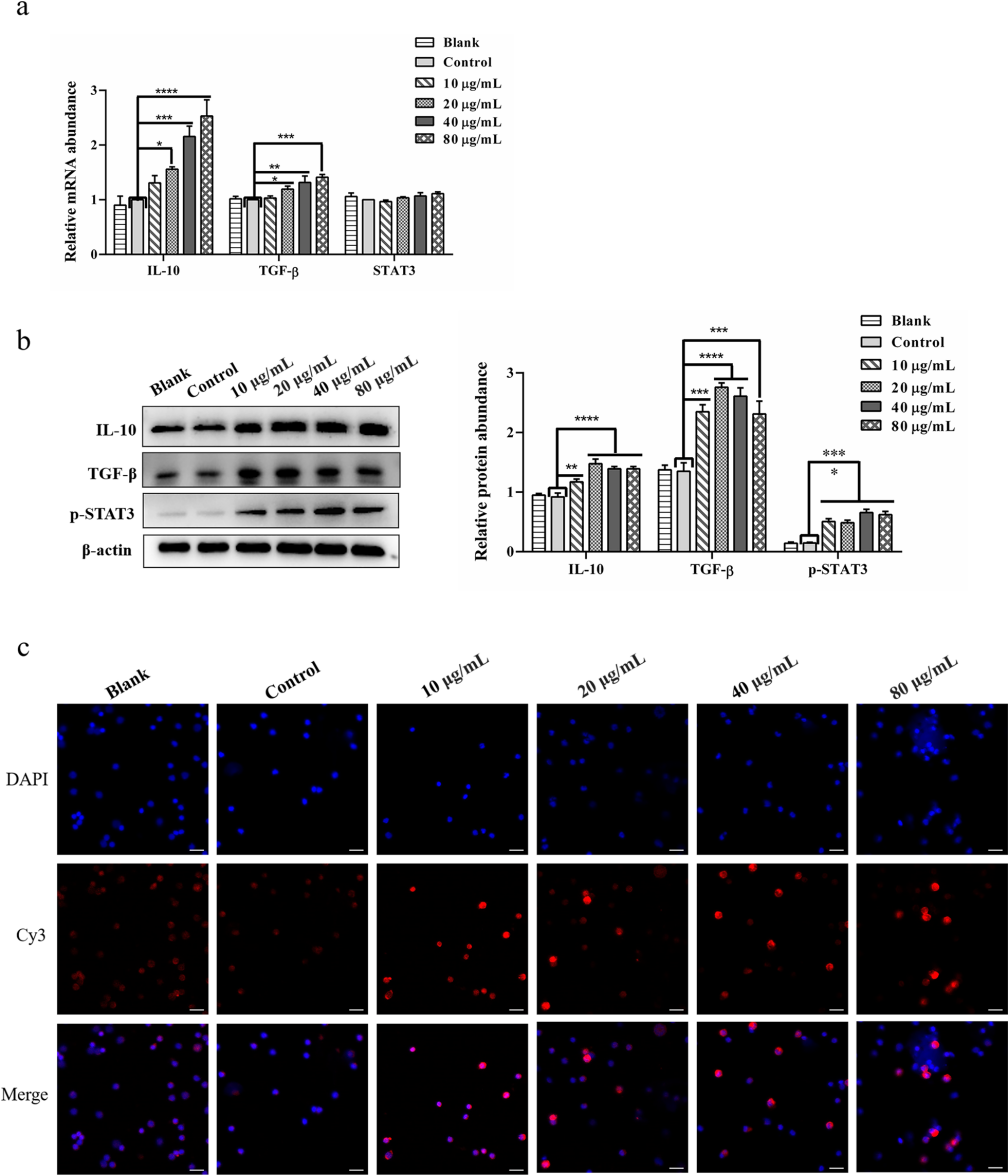
The rHcGOB protein activated the IL-10/TGF-β/STAT3 pathway in PBMCs. Cells were incubated with different concentrations of rHcGOB and with PBS (control) at 37 °C, and 5% CO_2_ for 72 h.** a** Transcription analysis of IL-10, TGF-β1, STAT3 in goat PBMCs. **b** The expression of IL-10, TGF-β1, p705-STAT3 was detected by western blot. The results presented here are representative of three independent experiments. Asterisks indicate significant differences at *P < 0.05, **P < 0.01, ***P < 0.001, and ****P < 0.0001 versus the control group. **c** Detection of nuclear translocation of phosphorylated STAT3 by IFA. The red color indicates localization of the target protein stained with Cy3 on PBMCs, and the blue color indicates the localization of nuclei stained with DAPI. Merge indicates the merged image of DAPI and Cy3. Scale bars: 20 μm

As indicated in Fig. 9, the in vitro incubation of PBMCs at various concentrations of rHcGOB protein (10, 20, 40, 80 μg/ml) significantly increased the expression of IL-10 (10 μg/ml: ANOVA, *F*_(5, 12)_ = 22.154, *P* = 0.0046; 20 μg/ml: ANOVA, *F*_(5, 12)_ = 22.154, *P* < 0.0001; 40 μg/ml: ANOVA, *F*_(5, 12)_ = 22.154, *P* < 0.0001; 80 μg/ml: ANOVA, *F*_(5, 12)_ = 22.154, *P* < 0.0001) and TGF-β (10 μg/ml: ANOVA, *F*_(5, 12)_ = 20.431, *P* = 0.0002; 20 μg/ml: ANOVA, *F*_(5, 12)_ = 20.431, *P* < 0.0001; 40 μg/ml: ANOVA, *F*_(5, 12)_ = 20.431, *P* < 0.0001; 80 μg/ml: ANOVA, *F*_(5, 12)_ = 20.431, *P* = 0.0003) (Fig. 9b). The incubation of PMBCs at 20, 40 and 80 μg/ml rHcGOB protein also significantly increased the mRNA transcript levels of IL-10 (10 μg/ml: ANOVA, *F*_(5, 12)_ = 15.005, *P* = 0.2169; 20 μg/ml: ANOVA, *F*_(5, 12)_ = 15.005, *P* = 0.0363; 40 μg/ml: ANOVA, *F*_(5, 12)_ = 15.005, *P* = 0.0004; 80 μg/ml: ANOVA, *F*_(5, 12)_ = 15.005, *P* < 0.0001) and TGF-β (10 μg/ml: ANOVA, *F*_(5, 12)_ = 8.163, *P* = 0.7229; 20 μg/ml: ANOVA, *F*_(5, 12)_ = 8.163, *P* = 0.0454; 40 μg/ml: ANOVA, *F*_(5, 12)_ = 8.163, *P* = 0.0032; 80 μg/ml: ANOVA, *F*_(5, 12)_ = 8.163, *P* = 0.0005) (Fig. 9a). The co-incubation of different concentrations of rHcGOB protein with PBMCs did not affect the transcript level of STAT3 (10 μg/ml: ANOVA, *F*_(5, 12)_ = 1.628, *P* = 0.5644; 20 μg/ml: ANOVA, *F*_(5, 12)_ = 1.628, *P* = 0.5319; 40 μg/ml: ANOVA, *F*_(5, 12)_ = 1.628, *P* = 0.2634; 80 μg/ml: ANOVA, *F*_(5, 12)_ = 1.628, *P* = 0.0743) (Fig. 9a), but did significantly increase the expression of phosphorylated STAT3 (p705-STAT3) (10 μg/ml: ANOVA, *F*_(5, 12)_ = 29.838, *P* = 0.0001; 20 μg/ml: ANOVA, *F*_(5, 12)_ = 29.838, *P* = 0.0001; 40 μg/ml: ANOVA, *F*_(5, 12)_ = 29.838, *P* < 0.0001; 80 μg/ml: ANOVA, *F*_(5, 12)_ = 29.838, *P* < 0.0001) (Fig. 9b). IFA showed that the different concentrations of rHcGOB protein significantly promoted STAT3 entry into the nucleus (Fig. 9c). These results suggest that rHcGOB activates the IL-10/TGF-β/STAT3 signaling pathway in PBMCs.

### Effect of rHcGOB on transcription and secretion of cytokines in PBMCs

**Fig. 10:**
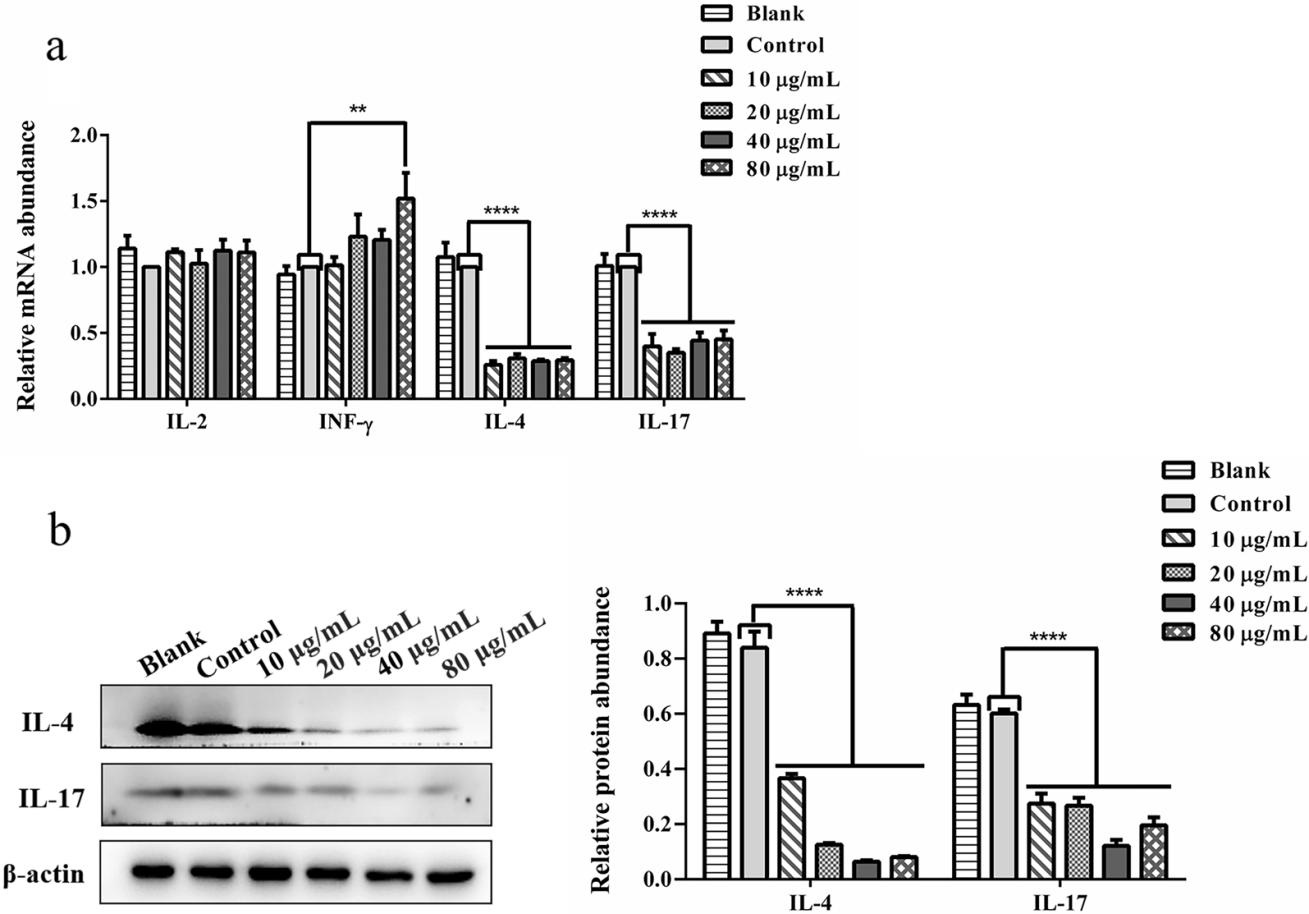
Effect of rHcGOB protein on the transcription and secretion of Th1-, Th2-, and Th17-related cytokines in PBMCs. Cells were incubated with different concentrations of rHcGOB protein and with PBS (control) for 24 h at 37 °C and 5% CO_2_. **a** Transcription analysis of INF-γ, IL-2, IL-4, IL-17 in goat PBMCs. **b** The expression of IL-4 and IL-17A was detected by western blot. The results presented here are representative of three independent experiments. Asterisks indicate significant differences at **P < 0.01, and ****P < 0.0001 versus the control group. Abbreviations: Th, T-helper cells

The different concentrations of rHcGOB (10, 20, 40, 80 μg/ml) significantly inhibited the transcription of IL-4 (10 μg/ml: ANOVA, *F*_(5, 12)_ = 63.991, *P* < 0.0001; 20 μg/ml: ANOVA, *F*_(5, 12)_ = 63.991, *P* < 0.0001; 40 μg/ml: ANOVA, *F*_(5, 12)_ = 63.991, *P* < 0.0001; 80 μg/ml: ANOVA, *F*_(5, 12)_ = 63.991, *P* < 0.0001) and IL-17 (10 μg/ml: ANOVA, *F*_(5, 12)_ = 22.391, *P* < 0.0001; 20 μg/ml: ANOVA, *F*_(5, 12)_ = 22.391, *P* < 0.0001; 40 μg/ml: ANOVA, *F*_(5, 12)_ = 22.391, *P* < 0.0001; 80 μg/ml: ANOVA, *F*_(5, 12)_ = 22.391, *P* < 0.0001), as well as the expression of IL-4 (10 μg/ml: ANOVA, *F*_(5, 12)_ = 160.797, *P* < 0.0001; 20 μg/ml: ANOVA, *F*_(5, 12)_ = 160.797, *P* < 0.0001; 40 μg/ml: ANOVA, *F*_(5, 12)_ = 160.797, *P* < 0.0001; 80 μg/ml: ANOVA, *F*_(5, 12)_ = 160.797, *P* < 0.0001) and IL-17 (10 μg/ml: ANOVA, *F*_(5, 12)_ = 54.869, *P* < 0.0001; 20 μg/ml: ANOVA, *F*_(5, 12)_ = 54.869, *P* < 0.0001; 40 μg/ml: ANOVA, *F*_(5, 12)_ = 54.869, *P* < 0.0001; 80 μg/ml: ANOVA, *F*_(5, 12)_ = 54.869, *P* < 0.0001) as shown in figures (Fig. 10a and b). However, the different concentrations of rHcGOB had no significant effect on the transcription of IL-2 (10 μg/ml: ANOVA, *F*_(5, 12)_ = 0.562, *P* = 0.3220; 20 μg/ml: ANOVA, *F*_(5, 12)_ = 0.562, *P* = 0.8172; 40 μg/ml: ANOVA, *F*_(5, 12)_ = 0.562, *P* = 0.2789; 80 μg/ml: ANOVA, *F*_(5, 12)_ = 0.562, *P* = 0.3292) in PBMCs (Fig. 10a). In contrast, the high concentration of 80 µg/ml of rHcGOB protein significantly promoted the transcription of INF-γ (10 μg/ml: ANOVA, *F*_(5, 12)_ = 3.433, *P* = 0.9299; 20 μg/ml: ANOVA, *F*_(5, 12)_ = 3.433, *P* = 0.1835; 40 μg/ml: ANOVA, *F*_(5, 12)_ = 3.433, *P* = 0.2314; 80 μg/ml: ANOVA, *F*_(5, 12)_ = 3.433, *P* = 0.0079) (Fig. 10a).

## Discussion

*Haemonchus contortus* is a nematode parasite that feeds on the blood of ruminants as a source of nutrition, causing serious effects and even death to grazing ruminants worldwide, especially to small ruminants [47, 48]. In this study, we cloned and expressed the gene encoding HcGOB, a key enzyme of the trehalose synthesis pathway of *H. contortus* and found that the rHcGOB protein was recognized by the sera from a goat infected with *H. contortus*. Immunofluorescence localization revealed a wide distribution of HcGOB protein in adult worms, with a strong positive expression in the microvilli of the worm intestine. This result strongly suggests that HcGOB protein can come into direct contact with and is more vulnerable to attack by host antibodies circulating in the peripheral blood. Based on our knowledge of the Barbervax® vaccine, the antigens are native integral membrane proteins isolated from the brush border of adult intestinal cells [5, 6]. The immunofluorescence localization results indicate that the Barbervax® antigens H-gal-GP and H11 are localized in the intestinal microvilli. These results suggest that the HcGOB protein of *H. contortus* possesses similar properties to both the H-gal-GP as well as H11 antigens that are readily exposed to attack by the host immune system.

*Teladorsagia circumcincta*, *Haemonchus placei* and *H. contortus* are all parasitic on the fourth gastric mucosa of ruminants, and all are acid tolerant and resistant to gastrin digestion [49, 50]. Multiple sequence alignment and phylogenetic analysis showed that the HcGOB gene is highly related to that of *T. circumcincta* and *H. placei*, respectively. The high homology of GOB genes among these three parasites may be closely related to their parasitic environment.

In nematodes, trehalose is thought to be a stress protector and source of energy, and to contribute to both glucose uptake and egg hatching [51]. Silencing of the TPP6 gene in *Serratia marcescens* by small interfering (si) RNA greatly impairs the process of early embryonic development, leading to the degradation of existing eggs and delaying further embryonic development [51]. The siRNA silencing of the GOB gene of *Caenorhabditis elegans* causes larval intestinal defects (manifested by an intestinal obstruction phenotype), leading to early larval lethality [28, 30]. Similarly, silencing of the TPP6 gene in *Mycobacterium tuberculosis* also leads to mycobacterial lethality [52]. The lethal effects of trehalose-6-phosphate have demonstrated in the nematode *Onchocerca volvulus*, in which it has been shown to be a better microfilaricidal and macrofilaricidal agent than diethylcarbamazine [53]. In our study, qPCR assays showed the transcription of the HcGOB gene at different developmental stages of the worm (eggs, iL3s, adults), revealing high levels of transcription in the xL3 and egg stages. These results suggest that trehalose may play an important role in embryo development and larval molting. Therefore, in a future study we are planning to explore the effects of silencing HcGOB on worm colonization, parasitism, growth and reproduction.

Previous studies have shown that various HcESP-derived protein molecules, such as rHcMTF-12 [54], rHCRD [37], rHc-GDC [40], rHcEF-1α [46], rHcES-15 [34], rMiro-1 [55], HCcyst-3 [56] and RHC-AK [57], can bind to PBMCs in vitro and regulate cellular immune function. Our studies showed that rHcGOB can bind to goat PBMCs and activate the IL-10/TGF-β/STAT3 pathway in PBMCs in vitro. Both IL-10 and IL-6 induce STAT3 activation, but with opposite effects; IL-6 stimulation promotes a proinflammatory response, but IL-10 signaling induces a strong anti-inflammatory response [58]. Recent studies have shown the critical contribution of the IL-10/STAT3 axis to the immune tolerance provided by Treg cells [59, 60], facilitating worm evasion from the host immune system. We noted that the rHcGOB protein also inhibited both the transcription and expression of IL-4, IL-17, but had no significant effect on the transcription of IL-2. The host showed mainly the Th2 type of immune response against helminth infection, characterized by the secretion of cytokines, such as IL-4, IL-5 and IL-13 [61–63]. It has been shown that anti-IL- 4 or anti-IL-4 receptor antibodies block the polyclonal IgE response to parasitic nematodes and block the host's immune protective mechanisms [64]. IL-17 is mainly secreted by Th17 cells, which promote IgA secretion and play an important role in host defense against pathogens at the mucosal barrier [65]. Our data suggest that rHcGOB activates Treg cells to exert anti-inflammatory effects while inhibiting IL-4 secretion by Th2 cells and IL-17 secretion by Th17 cells. HcGOB may be a key molecule for the occurrence of immune evasion in *H. contortus*, suggesting that the sequestration of HcGOB by antibodies can be expected to be a new strategy for the control of haemonchosis.

## Conclusions

In conclusion, the HcGOB gene was transcribed at all life-cycle phases of *H. contortus* and was found to be highly expressed in the intestine at the adult stage. It was also localized around the embryos of female adult worms. This result indicates that the HcGOB gene could be a promising candidate for vaccine development against *H. contortus.* The rHcGOB protein activated the IL-10/TGF-β/STAT3 pathway in PBMCs while inhibiting Th2 and Th17 responses; this result might reveal a new mechanism for parasite immune evasion.

## Supplementary Information

**Additional file 1: Table S1.** Primers used to amplify the HcGOB gene. **Table S2.** Primers used for qPCR experiments.

**Additional file 2: Figure S1**. Transmembrane structure prediction using TMHMM Server v.2.0. The amino acid sequences of HcGOB (NCBI accession number: HF967182.1) was analyzed to predict transmembrane structures using TMHMM Server v.2.0. No transmembrane domains were predicted in this protein structure (http://www.cbs.dtu.dk/services/TMHMM/). **Figure S2.** Signal peptide prediction. The amino acid sequences of HcGOB (NCBI Accession Numbers: HF967182.1) was used to predict signal peptides by SignalP 4.1Server. No signal peptides were predicted in this protein structure ( http://www.cbs.dtu.dk/services/SignalP/). **Figure S3.** N-glycosylation site prediction. The amino acid sequences of HcGOB (NCBI accession number: HF967182.1) was used to predict N-glycosylation site by the NetNGlyc 4.1 Server. The predicted results showed the presence of an N-glycosylation site in the protein structure (http://www.cbs.dtu.dk/services/NetNGlyc/).

## Data Availability

All data generated or analyzed during this study are included within the article and its Additional files 1, 2.

## Abbreviations

BSA: Bovine serum albumin
CCK-8: Cell counting kit-8
cDNA: Complementary DNA
Cy3: Sulfo-Cyanine 3
DAPI: 4',6-Diamidino-2-phenylindole
HcGOB: *Haemonchus contortus* GOB protein
HRP: Horseradish peroxidase
IFA: Immunofluorescence assay
IFN-γ: Interferon gamma
IL: Interleukin
iL3s: Infective third-stage larvae
IPTG: Isopropyl-β-D-thiogalactopyranoside
LB: Luria–Bertani
Ni-NTA: Ni2^+^-nitrilotriacetic acid
NO: Nitric oxide
PBMCs: Peripheral blood mononuclear cells
PBS: Phosphate-buffered saline
PBST: PBS containing 0.1% Tween 20
PVDF: Polyvinyl difluoride
qPCR: Quantitative real-time PCR
STAT3: Signal transducer and activator of transcription 3
TBS-T: Tris-buffered saline containing 0.05% Tween-20
TGF-β: Transforming growth factor β
TPP6/GOB: Trehalose-6-phosphate phosphatase
xL3s: Exsheathed third‑stage larvae
