# Supplementary material for: In vitro characterization of Haemonchus contortus trehalose-6-phosphate phosphatase and its immunomodulatory effects on peripheral blood mononuclear cells (PBMCs)
Source: Parasit Vectors. 2021 Dec 20;14:611. doi: 10.1186/s13071-021-05115-4 (PMC8685816; doi:10.1186/s13071-021-05115-4)
Supplement: Supplementary file 1 — Additional file 1: Table S1. Primers used to amplify the HcGOB gene. Table S2. Primers used for qPCR experiments. [file 13071_2021_5115_MOESM1_ESM.docx]

**Table S1. Primers used to amplify the HcGOB gene**

| **Target genes** | **Primer sequences (5’-3’)** | |
| --- | --- | --- |
| GOB | Forward primer | CGGGATCCATGTCACCCGGGAGCGG |
|  | Reverse primer | CCCAAGCTTCTAATCCAGTTCAACTTTTGCAGCCA |

Note: The underlined sequences are the *BamH*I and *Hind*III digestion sites.

**Table S2. Primers used for qPCR experiments**

| **Target genes** | **Forward primer (5′-3′)** | **Reverse primer (5′-3′)** | **Product size (bp)** |
| --- | --- | --- | --- |
| β-actin | CACCACACCTTCTACAAC | TCTGGGTCATCTTCTCAC | 106 |
| IL-2 | CAAACGGTGCACCTACTTCA | AGCTTGAGGTTCTCGGGATT | 115 |
| IL-4 | GTACCAGCCACTTCGTCCAT | GCTGCTGAGATTCCTGTCAA | 148 |
| IL-10 | CCTTGTCGGAAATGATCCAG | AGGGCAGAAAACGATGACAG | 150 |
| IL-17 | TTGTAAAGGCAGGGGTCATC | GGTGGAGCGCTTGTGATAAT | 149 |
| TGF-β | GAACTGCTGTGTTCGTCAGC | TCCAGGCTCCAGATGTAAGG | 126 |
| IFN-γ | GAACGGCAGCTCTGAGAAAC | GGTTAGATTTTGGCGACAGG | 131 |
| STAT3 | GAGAAGGACATCAGCGGCAAGAC | AGATAGACCAGCGGAGACACCAG | 146 |
| HcGOB | GACGGTGTGGCGGCATTGATAG | ATTGGCAAGTCGCTGGTGGTATC | 92 |
| β-tubulin | TGCTATGTTCCGTGGTCGTATG | CGGCAGTCTTAACGTTGTTTGG | 116 |
