# Supplementary material for: In vitro characterization of Haemonchus contortus trehalose-6-phosphate phosphatase and its immunomodulatory effects on peripheral blood mononuclear cells (PBMCs)
Source: Parasit Vectors. 2021 Dec 20;14:611. doi: 10.1186/s13071-021-05115-4 (PMC8685816; doi:10.1186/s13071-021-05115-4)
Supplement: Supplementary file 2 — Additional file 2: Figure S1. Transmembrane structure prediction using TMHMM Server v.2.0. The amino acid sequences of HcGOB (NCBI accession number: HF967182.1) was analyzed to predict transmembrane structures using TMHMM Server v.2.0. No transmembrane domains were predicted in this protein structure (http://www.cbs.dtu.dk/services/TMHMM/). Figure S2. Signal peptide prediction. The amino acid sequences of HcGOB (NCBI Accession Numbers: HF967182.1) was used to predict signal peptides by SignalP 4.1Server. No signal peptides were predicted in this protein structure ( http://www.cbs.dtu.dk/services/SignalP/). Figure S3. N-glycosylation site prediction. The amino acid sequences of HcGOB (NCBI accession number: HF967182.1) was used to predict N-glycosylation site by the NetNGlyc 4.1 Server. The predicted results showed the presence of an N-glycosylation site in the protein structure (http://www.cbs.dtu.dk/services/NetNGlyc/). [file 13071_2021_5115_MOESM2_ESM.pdf]

TMHMM result

```
# WEBSEQUENCE Length: 432
# WEBSEQUENCE Number of predicted TMHs:  0
# WEBSEQUENCE Exp number of AAs in TMHs: 0.0252
# WEBSEQUENCE Exp number, first 60 AAs:  0
# WEBSEQUENCE Total prob of N-in:        0.00259
WEBSEQUENCE     TMHMM2.0        outside      1   432
```

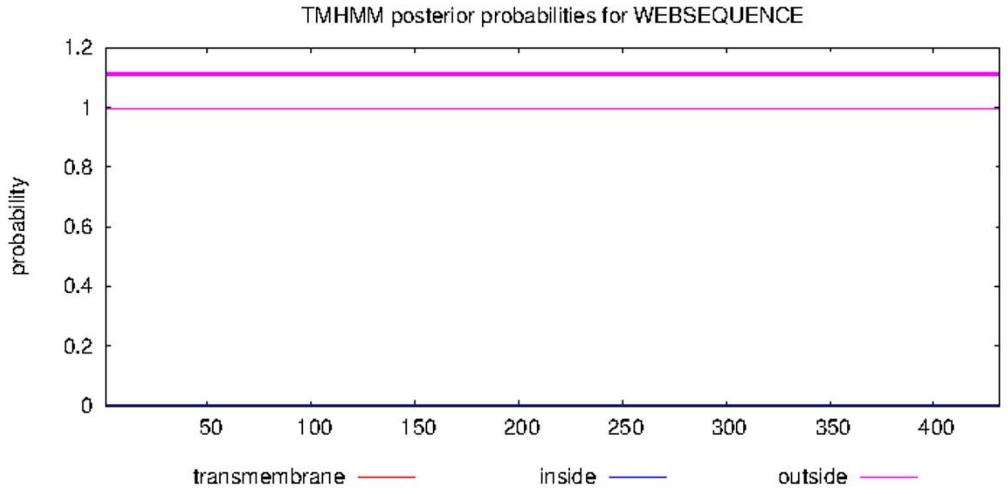

Figure S1. Transmembrane structure prediction using TMHMM Server v.2.0. The amino acid sequences of HcGOB (NCBI accession numbers: HF967182.1) was analyzed to predict transmembrane structures using TMHMM Server v.2.0. There were no transmembrane domains was predicted in this protein structure. http://www.cbs.dtu.dk/services/TMHMM/.

Predictions list. Use the help page for more detailed description of the output page.

VERSITY OF DENMARK DTU

Predicted proteins

Sequence
Prediction: Other

Protein type	Signal Peptide (Sec/SPI)	Other
**Likelihood**	0.0012	0.9988

Download: PNG / EPS / Tabular

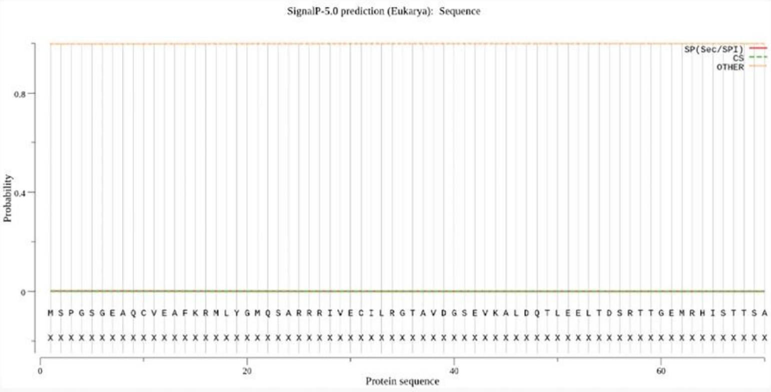

Figure S2. Signal peptide prediction. The amino acid sequences of HcGOB (NCBI accession numbers: HF967182.1) was used to predict Signal peptides by SignalP 4.1Server. There were no Signal peptides was predicted in this protein structure. http://www.cbs.dtu.dk/services/SignalP/.

NetNGlyc 1.0 Server - prediction results

Technical University of Denmark

Asn-Xaa-Ser/Thr sequons in the sequence output below are highlighted in **blue**.
Asparagines predicted to be N-glycosylated are highlighted in **red**.

Output for 'Sequence'

```
##########################################################################################

    Warning: This sequence may not contain a signal peptide!!

    Proteins without signal peptides are unlikely to be exposed to
    the N-glycosylation machinery and thus may not be glycosylated
    (in vivo) even though they contain potential motifs.

    SignalP-NN euk predictions are as follows:

  # name                    Cmax  pos ?  Ymax  pos ?  Smax  pos ?  Smean ?  D     ?
  Sequence                       0.108  12  0.118  12  0.143   1  0.113   0.115 N  0.450      SignalP-noTM

    SignalP output is explained at http://www.cbs.dtu.dk/services/SignalP/output.html

##########################################################################################

Name:  Sequence          Length:  432
MSPGSGEAQCVEAFKRMLYGMQSARRRIVECILRGTAVDGSEVKALDQTLEELTDSRTTGEMRHISTTSADFPINIRDEI      80
RGLRKDCEFLHRLSQHESGTIEHILEGLSLETILAPYHPNGPEKFHEELLNAEQFLMGFVDSADTGIKPLLVTDWDGTMK     160
DYCSQYATNLQPVYSAVVMGRFAELFTRATAVLTAGPLRGPGILDLTALPINGPVMFSGSWGREWWLRGRRVVHDDGISE     240
EGFDAIGRLSDEMTDLLANGVFSQFALVGSGVQRKVDRLTLGVQTVFGHVPLELVVRYIEAVKERIHRVDPNNTSLVLET     320
SSPLEIEVCVHNSGAVWNKGDGVAALIESLHDTLKNGKVLVAGDTTSDLPMLQHAVSENRTGAMALFVGAGDSLRESVRS     400
IVGDDSRICFVSCPDVVHAAFARILAAKVELD
................................................................................      80
................................................................................     160
................................................................................     240
................................................................................     320
..........................................................N.....................     400
................................                                                     480

(Threshold=0.5)
```

SeqName	Position	Potential	Jury agreement	N-Glyc result
Sequence	312 NNTS	0.4667	(7/9)	-
Sequence	313 NTSL	0.4421	(7/9)	-
Sequence	379 NRTG	0.5062	(4/9)	+

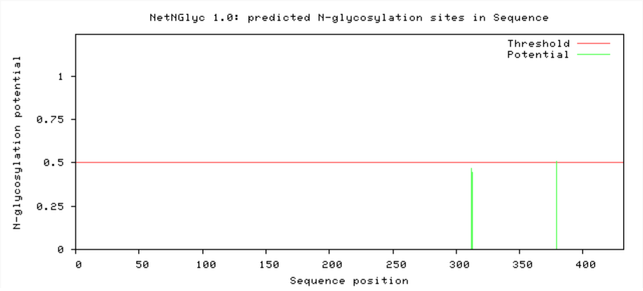

Figure S3. N-glycosylation site prediction. The amino acid sequences of HcGOB (NCBI accession numbers: HF967182.1) was used to predict N-glycosylation site by NetNGlyc 4.1 Server. The predicted results showed the presence of an N-glycosylation site in the protein structure. http://www.cbs.dtu.dk/services/NetNGlyc/#opennewwindow.
